# Comment on: A single dose recombinant AAV based CHIKV vaccine elicits robust and durable protective antibody responses in mice

**DOI:** 10.1371/journal.pntd.0013669

**Published:** 2025-11-20

**Authors:** Shenghao Xu, Kaiyu Li, Andres Merits, Zimeng Dai, Jie Luo, Xiangli Zhong, Ouyang Zhang, Jian Yang, Xiaoyao Yang

**Affiliations:** 1 Department of Clinical Medicine, North Sichuan Medical College, Nanchong, People’s Republic of China; 2 Institute of Basic Medicine, North Sichuan Medical College, Nanchong, People’s Republic of China; 3 Institute of Bioengineering, University of Tartu, Tartu, Estonia; 4 School of Sports Medicine and Rehabilitation, North Sichuan Medical College, Nanchong, People’s Republic of China; Colorado State University, UNITED STATES OF AMERICA

Zhu et al. [1] developed a recombinant adeno-associated virus serotype 1 (rAAV1) vector-based CHIKV vaccine candidate that encodes the full structural polyprotein of chikungunya virus (CHIKV) and demonstrated that it induces a strong and long-lasting antibody response in mice. Based on this, it was expected that the AAV vector can be utilized to develop a vaccine against mosquito-borne alphaviruses. While the results of the study are compelling, several points need to be further discussed to determine the translational potential of this platform and answer outstanding questions.

## Pre-existing immunity to AAV: A translational barrier

The predominant challenge to AAV vector-based vaccines is presented by the pre-existing immunity to AAV capsid proteins. Seroprevalence studies have yielded evidence of neutralizing antibodies (Nabs) to AAV1 being present in approximately 30–70% of the world’s population, with varying levels based on geographic region and age [2]. This variation depends on the incidence of wild-type AAV infection and prior exposure to AAV-based treatments. Pre-existing immunity can interfere with vaccine efficacy through the prevention of viral vector transduction or altered immune clearance by opsonization [3]. Zhu et al. overcame this hurdle by employing a heterologous prime-boost regimen (rAAV1 prime and rAAV9 boost), a strategy used in some adenovirus-based COVID-19 vaccines. Though such a heterologous prime-boost strategy reduces vector-specific NAb interference, it may not eliminate the problem. Cross-reactive pre-existing immunity to many AAV serotypes is a prevalent phenomenon owing to conserved epitopes in the capsid shared among the AAVs. For example, individuals with anti-AAV2 NAbs cross-neutralize also AAV3, AAV5, and AAV6 due to shared structural motifs within the VP3 domain [4]. This phenomenon creates concerns about whether serotype-switching strategies can efficiently counteract the general anti-AAV immunity in human population. Furthermore, available evidence indicates that potent immunogenicity elicited by viral vectors in rodents or NHPs frequently contracts to modest or transient antibody responses when translated to human doses [5–7].

To address this translational barrier, future studies should systematically evaluate the impact of pre-existing anti-AAV immunity in animal models. Ex-vivo neutralization assays using human or mouse sera would allow quantitative evaluation of the NAb titer threshold that negates vaccine efficacy [8]. Additionally, engineering AAV capsids with “stealth” modifications—such as PEGylation or incorporation of non-natural amino acids—could reduce antigenicity while maintaining transduction efficiency [9].

## The role of cellular immunity in protection

While Zhu et al. provide robust humoral data, extending the analysis to include T-cell responses would offer a more complete picture of antiviral immunity. Agarwal et al. [10] recently showed that CHIKV-specific CD4 ⁺ T cells correlate with chronic arthritic symptoms in humans, underscoring that joint pathology is driven by T-cell–mediated inflammation rather than viral persistence. Broeckel et al. [11] further demonstrate that CD8 ⁺ T cells can ameliorate chronic arthritis in mice, even when viraemia is already antibody-controlled. Including CD8⁺ or IFN-γ readouts in future analyses would therefore reveal whether the rAAV1 vaccine simply suppresses viraemia or also dampens the T-cell–dependent joint disease that determines long-term morbidity.

The inclusion of orthogonal assays for the assessment of cellular immunity would increase the strength of the study. T-cell correlates of protection should be mapped by brief ex-vivo assays and depletion/passive-transfer studies to complement the current antibody-focused readouts. Such results are especially crucial for comparisons of the rAAV1-CHIKV-SP with other vaccine platforms. For example, the measles virus-vectored CHIKV vaccine (MV-CHIKV) elicits robust T-cell responses but requires cold-chain storage, while aluminum-adjuvanted subunit vaccines predominantly drive antibody responses [12]. AAV vectors, with their sustained antigen expression, may uniquely prime both arms of adaptive immunity, but empirical validation is needed.

## Dose optimization and scalability

Population-scale CHIKV prophylaxis would demand 10²⁴ genome copies (GC) of rAAV1-CHIKV-SP—equivalent to >10⁶ L of high-density rAAV production cell culture and orders of magnitude above the ≈ 2 × 10¹⁵ GC yr ⁻ ¹ global vaccine-grade AAV capacity projected for 2025 [13]. Current vaccine suites (≈2 × 10³ L bioreactors) would require >500 parallel runs annually—far beyond routine GMP scheduling. Dose-sparing strategies could enhance feasibility of the approach. Intramuscular electroporation, which increases cellular uptake of AAV vectors, has boosted transgene expression 10-fold in macaque studies [14].

At these GC levels, the high-dose AAV “class effect” safety profile observed in gene-therapy cohorts is also expected to re-emerge. Haemophilia and SMA recipients, receiving 2 × 10¹³ GC kg ⁻ ¹, have experienced transient ALT > 2.5 × ULN, γ-GT rise, C3a/C5a elevation, complement consumption–induced hypoalbuminaemia, rare thrombotic microangiopathy with schistocytes, hypertension and proteinuria, as well as infusion-related fever, chills and dose-dependent innate cytokine release [15]. Consequently, first-in-human AAV1-CHIKV protocols must incorporate on-day-1 and week-4 monitoring of hepatic enzymes, total complement, platelet counts, blood pressure, urinalysis and peripheral blood smear. Application of these surveillance standards used for small-cohort gene-therapy settings for much larger scale vaccine trials would be challenging.

Furthermore, current studies were limited to 12-week follow-up monitoring period, raising concerns about potential long-term efficacy and tolerance. Long-term surveillance of neutralizing antibody titers and memory B-cell kinetics will clarify whether rAAV1-CHIKV-SP can maintain durable protection [1].

## The breadth of protection against heterologous strains

The vaccine’s cross-protection against the East/Central/South African (ECSA) strain of CHIKV is encouraging. However, while E2 glycoproteins of CHIKV genotypes and clades display high degree of conservation, the recent emergence of the ECSA Indian Ocean Linage (IOL) sub-clade carrying the E2-K233R substitution highlights a potential antigenic drift risk [16]. Zhu et al. used whole-virion ELISA to quantify neutralization; this assay (i) presents a fixed virion surface that masks variable E2-E1 epitopes [17], (ii) cannot detect escape caused by E2-K233R or mutations located at E2-E1 interface, and (iii) yields data showing bulk absorbance rather than reduction of infectious (plaque-forming) units. Taken together, these factors may result in over-estimation of cross-neutralization efficacy against Asian or IOL strains that harbour E2-K233R or altered glycosylation of envelope proteins [18]. Replacing whole-virion ELISA with pseudovirus assays or with plaque reduction neutralization test (PRNT) would allow detection of epitope-specific escape and provide neutralization titers required for regulatory cross-clade dossiers.

In response to this, future studies would have to conduct neutralization tests against a globally representative panel of CHIKV strains, i.e., Asian (e.g., Singapore 2015; Asian/Caribbean), West African (e.g., Senegal 1983), ECSA-American (e.g., RJ1) and contemporary IOL isolates. Furthermore, closely related o’nyong’nyong virus that has high antigenic similarity with CHIKV should be included. Structural vaccinology approaches, such as cryo-EM mapping of vaccine-elicited antibodies bound to heterologous E2-E1 heterodimers, could identify conserved neutralizing epitopes. This is particularly urgent in light of the partial protection by IxChiq (VLA1553) against African strains in clinical trials because of E2-E1 glycosylation pattern diversity [19].

## Conclusion

In a mouse model, rAAV1-CHIKV-SP achieves durable, high-titer neutralization with a single dose. However, to enhance the future applicability of this vaccine, several aspects of immune protection warrant further consideration, including the impact of pre-existing AAV1 antibodies, analysis of T-cell responses, optimization of the dosing regimen—as the 5 × 10¹⁰ GC benchmark lies on a still-rising dose–response curve—and validation of cross-genotype efficacy by providing robust PRNT data using both historical and contemporary CHIKV isolates, including E2-K233R IOL variants. By addressing these factors, the single-dose, long-lasting, and broad-spectrum protection observed in mice by Zhu and colleagues could ultimately be translated to humans.
